# RCS Diversity of Electromagnetic Wave Carrying Orbital Angular Momentum

**DOI:** 10.1038/s41598-017-15250-7

**Published:** 2017-11-13

**Authors:** Chao Zhang, Dong Chen, Xuefeng Jiang

**Affiliations:** Labs of Avionics, School of Aerospace Engineering, Tsinghua University, Beijing, 100084 P. R. China

## Abstract

An electromagnetic (EM) wave with orbital angular momentum (OAM) has a helical wave front, which is different from that of the plane wave. The phase gradient can be found perpendicular to the direction of propagation and proportional to the number of OAM modes. Herein, we study the backscattering property of the EM wave with different OAM modes, i.e., the radar cross section (RCS) of the target is measured and evaluated with different OAM waves. As indicated by the experimental results, different OAM waves have the same RCS fluctuation for the simple target, e.g., a small metal ball as the target. However, for complicated targets, e.g., two transverse-deployed small metal balls, different RCSs can be identified from the same incident angle. This valuable fact helps to obtain RCS diversity, e.g., equal gain or selective combining of different OAM wave scattering. The majority of the targets are complicated targets or expanded targets; the RCS diversity can be utilized to detect a weak target traditionally measured by the plane wave, which is very helpful for anti-stealth radar to detect the traditional stealth target by increasing the RCS with OAM waves.

## Introduction

Electromagnetic (EM) waves contain angular momentum, which is composed of spin angular momentum and orbital angular momentum (OAM)^1^. For a radio wave, i.e., the EM wave in radio frequency, the spin angular momentum corresponds to polarization, which has already been widely used in radar applications^2^. However, OAM has not yet been applied to radar detection and is seldom discussed^3^. The main difference between the OAM wave and the plane wave is that the OAM wave has a spatial wave front, with the circle phase gradient in the transverse section perpendicular to the propagation direction of the wave^4^. This phase difference can be denoted as *e* ^*ilϕ*^, which depends on the azimuthal angle *ϕ* and the OAM mode number *l*. Whether or not this phase gradient can be utilized for improving the detection capability becomes the fundamental problem of the feasibility of OAM waves applied in radar applications.

Originally, OAM was employed in optical fibre transmission. Due to the orthogonality of OAM lights with co-axial reception, different OAM waves can be transmitted and received in the same frequency^5^. Thus, spectrum efficiency and the transmission capacity can be tremendously increased. OAM in radio wave is explored in free-space radio communications with its potential multiplexing. In 2007, B. Thidé first performed radio wave simulation and proposed the constraints of generating OAM by using an antenna array^6^. In 2011, Tamburini conducted the first transmission experiment with 442 m to verify OAM radio wave multiplexing and spectrum efficiency improvement^7^. In 2016, an experiment with the range of 27.5 km in China was successfully carried out to show the capability of OAM transmission in long distance^8^. In radar applications, preliminary discussions of the feasibility and application form of the OAM wave have lagged. In 2013, Guo proposed that an OAM wave has the potential of acquiring the cross-range profile of the target^9^. In 2015, Liu extended this property and proposed a two-dimensional radar imaging method^10^. Subsequently, he used power spectrum estimation algorithms and pulse-compression technology to increase the imaging resolution^11^. In 2017, a high-resolution radar imaging scheme was proposed with experiments^12^. These studies focused on the orthogonality of OAM waves and made use of OAM as a new dimension for radar image resolution improvement. However, the spatial phase difference on the plane perpendicular to the wave propagation has not yet been considered.

In the case that the OAM wave is scattered by a complicated target, due to the spatial phase difference of the incident wave, the backscattered wave is definitely not the same as that of the plane wave. Therefore, the radar cross section (RCS) of the target is expected to be different among OAM waves carrying different mode numbers. In this study, an experiment is designed to show the RCS difference of the backscattered OAM waves and the traditional plane wave. The RCS diversity capability of the OAM waves is revealed, which consolidates the radar application for stealth target detection.

## Results

### RCS measurement facility for OAM waves

**Figure 1:**
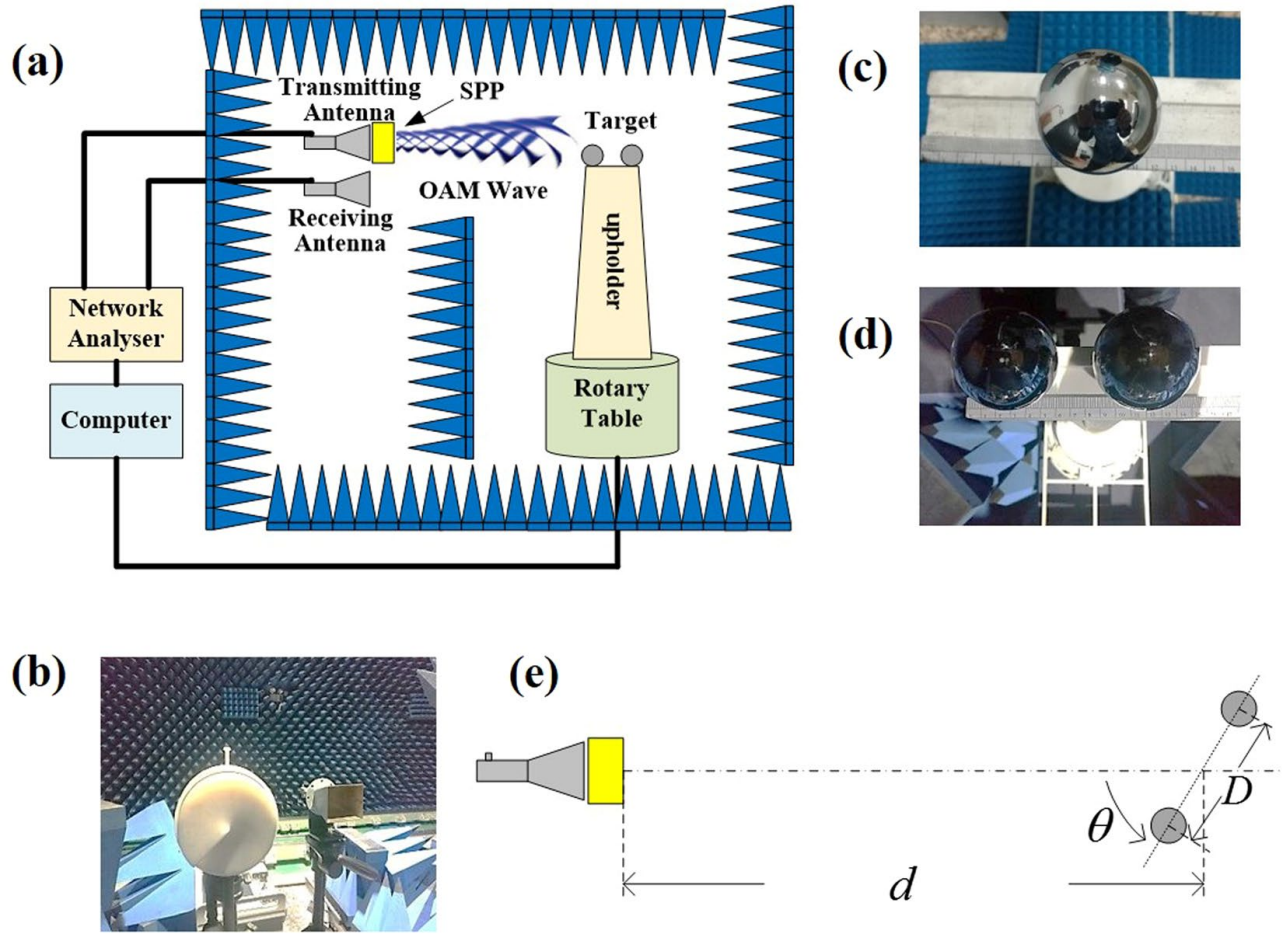
Experiment scenario for RCS measurement. (**a**) The system structure. (**b**) The transmitter with SPP. (**c**) The target with a single metal ball. (**d**) The target with two metal balls. (**e**) The conceptual figure with notations.

The common definition of RCS can be found in previously published studies^13,14^. Figure 1 shows the experimental facility for the RCS measurement. Figure 1a shows the structure of the whole experimental system. A spiral phase plate (SPP) with horn antenna is used to generate OAM wave (Fig. 1b). The OAM wave irradiates on a target, which is composed of a single metal ball as a simple target (Fig. 1c) and two metal balls as a complicated target (Fig. 1d). The target is deployed on a rotary table so that the RCS can be measured in 360°. Another horn antenna is used to receive the backscattered wave, which is confined to far-field regulation^15^. For radar detection, it is not necessary to identify the signals of OAM waves, i.e., only the electric field from the receiving signal is required. Unlike the transmitting end, the horn antenna at the receiving end does not need to be covered with SPP. Different from the co-axial receiving in OAM wave communications, due to the signal reflection and scattering from only a small part of the phase plane incident to the target, the whole spiral phase plane cannot be received at the receiving end for radar detection. Then, the backscattered signal from a small part of the spiral phase plane can be regarded as the signal carried by the plane wave^10^.

In the experiment as shown in Fig. 1, the target as well as the transmitting and receiving antennas are located in the same horizontal plane. In the case of a complicated target, the two balls are symmetrically located on the upholder. Due to the symmetry of RCS, the angle *θ* varying from 0° to 180° is enough. As shown in Fig. 1e, the distance between the target and the antennas is denoted by *d*, and the size of the target is denoted by *D*. In the measurement, we set *D* = 0.12 m and *d* = 3 m. The frequency is set as 35 GHz for two major reasons: 1) many high-precision airborne guidance systems work under this frequency for target detection, especially for stealth targets in tactics; 2) due to the small size and mature commercial off-the-shelf (COTS) products, the experimental equipment for 35 GHz in millimetre waves can be easily ordered and is more conveniently deployed. The parameters satisfy the far-field condition^14^, i.e., $d\ge 2\frac{{D}^{2}}{\lambda }$. The transmitted and received signals correspond to Port 1 and Port 2 of the network analyser (Agilent PNX 5244 A). A computer is employed to control the network analyser and the rotary table.

### Experiment 1: Simple target with single metal ball

**Figure 2:**
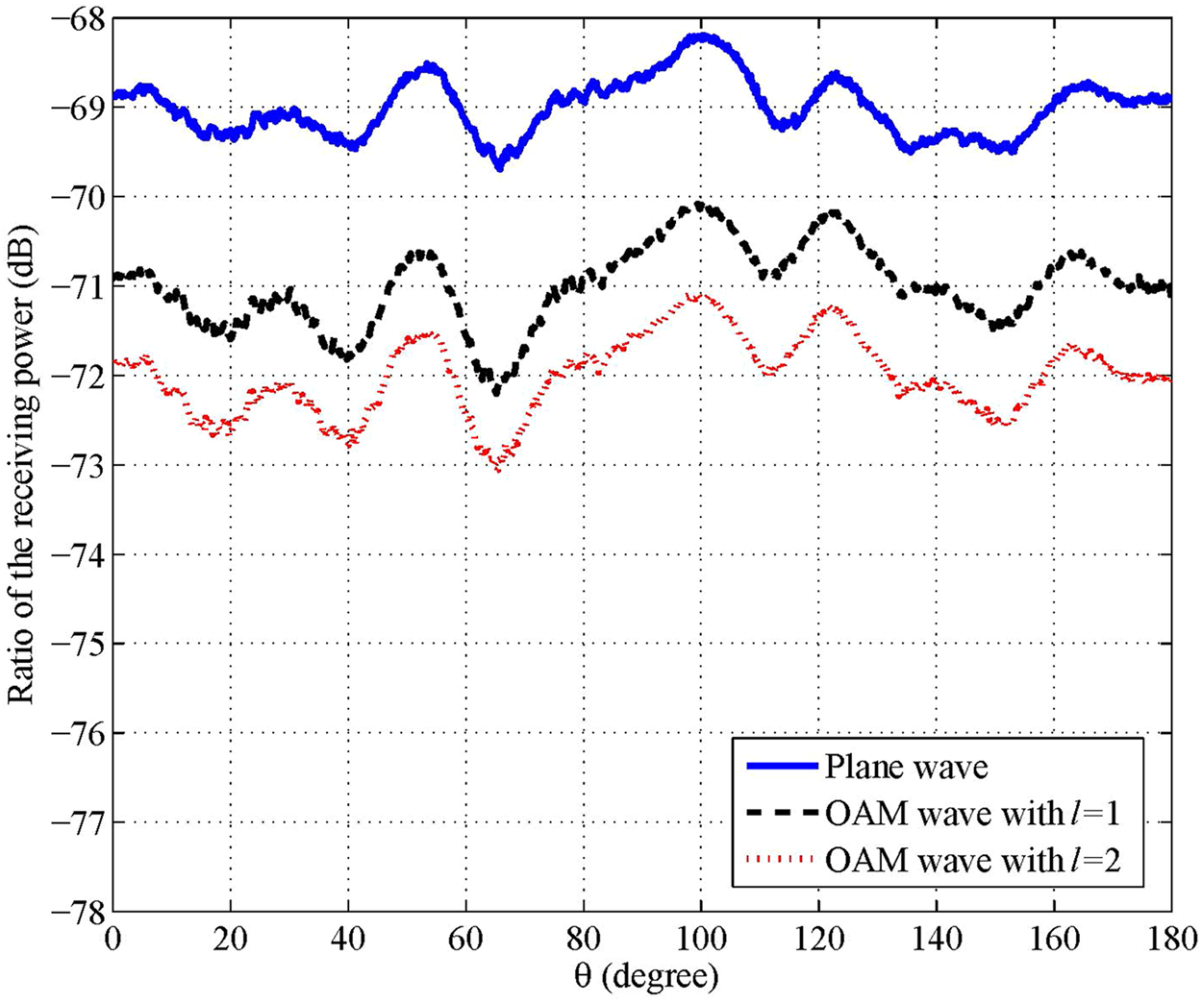
Power ratio of signals in the two ports of the network analyser for a single ball target. The power of the transmitted wave is 10 dBm, the radius of the ball is 2.5 cm, the antenna gain is 22.1 dBi, the distance between antenna and the target is 3 m, the resolution of the rotary table is 0.1°, and the antennas and the target have the same height of 82 cm.

Usually, the simple target with a single ball can be employed for initial calibration for RCS measurement. In our experiment, the single metal ball has a radius of *r* = 2.5 cm with the standard RCS of −27 dBsm. Before measuring the backscattered electromagnetic wave from the metal balls, a calibration measurement was conducted to test if the background noise satisfies the requirement of the standard RCS measurement. The backscattered wave from the rotary table is non-negligible interference for the RCS measurement. In our experiment, after wrapping the rotary table with wave absorbing material to block the interference sources, the experimental environment satisfies the RCS measurement requirements^14^, i.e., when the rotary table rotates, the received signal power varies within 1 dBm; this power is 20 dBm lower than that with a metal ball on the table. The background level is controlled below −48 dBsm in the microwave anechoic chamber. The scattered signal is approximately 20 dB larger than the background level. We record the power ratio of two ports of the network analyser to identify the backscatter power. Figure 2 compares the experimental results among the traditional plane wave and the OAM waves with mode numbers *l* = 1 and *l* = 2. The following is observed:

1. For the three cases, the variances of the receiving power ratio are almost within ±1 dB, which confirms the validity of the environment of the microwave chamber and the experimental values for further calibration. This can also be shown by comparison with the experiment and simulation of the calibrated RCS calculated from Fig. 2 (see Figure S1 in the Supplementary Information).
2. The curves of the three cases are similar, which means the OAM waves for the simple target in far-field detection have the same backscattered results as the plane wave. The plane wave can also be considered as an OAM wave with *l* = 0. This resolves the doubt whether the OAM wave is equivalent to the plane wave for a simple target, which has only one scatter point in the far field.

### Experiment 2: RCS for a complicated target

**Figure 3:**
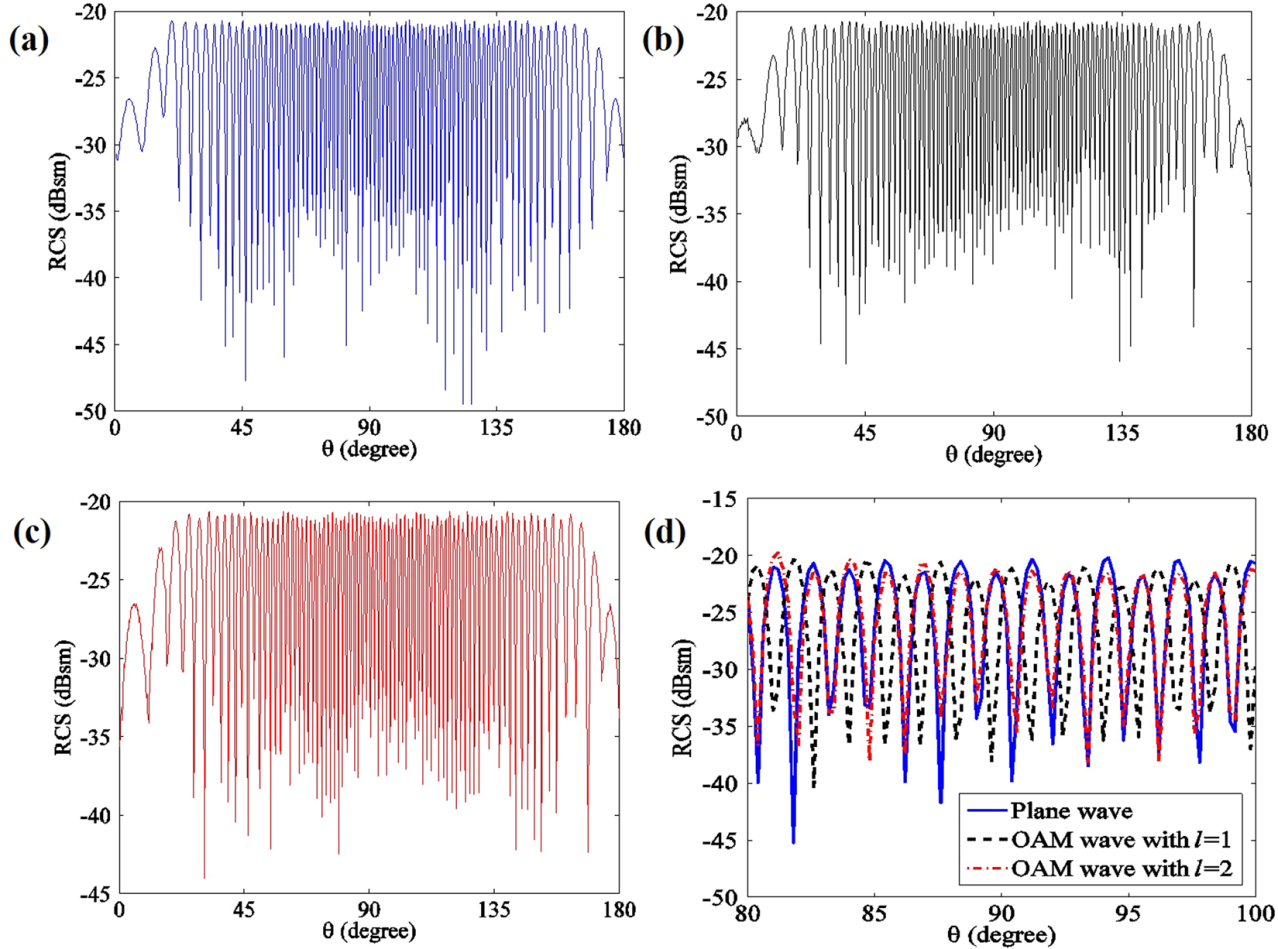
RCS of OAM waves with different mode number *l*. (**a**) RCS of the plane wave, i.e., OAM wave with *l* = 0. (**b**) RCS of the OAM wave with *l* = 1. (**c**) RCS of the OAM wave with *l* = 2. (**d**) Detailed comparison of the three waves.

Calibrated by the single-ball scattered power ratio, the RCS can be measured. For details of the procedures, refer to the reference^16^. A common target should usually be handled as a complicated target with multiple scatter points rather than an ideal simple target with only one scatter point. In the case of a complicated target, we employed the two-ball scenario. In the experiment, two isotropy scatters (metal balls) are located with *d* = 3 m away from the transmitting antenna, at the frequency of 35 GHz; this distance satisfies the far field condition with $d > 2\frac{{D}^{2}}{\lambda }$, where *D* is the maximum size of the target. Here, *D* = 0.12 m is defined as the distance between the centres of the two metal balls. The simulation result is shown in Fig. 3. Figure 3a shows the RCS of the plane wave, Figure 3b is the RCS of the OAM wave with *l* = 1, and Figure 3c is the RCS of the OAM wave with *l* = 2. To clearly highlight the details, Figure 3d presents the comparison among plane waves, with OAM waves with *l* = 1 and *l* = 2 from 80° to 100°. From these figures, we can observe the following:

1. For RCS from 0° to 180°, the first two peaks and the last two peaks are smaller than the rest of the peaks because one ball is sheltered from the other approximately 0° to 180°. The minimum peak value is −27.1 dBsm, which is almost equal to the RCS of a single ball. The values of the rest of the peaks are approximately −21 dBsm.
2. According to Fig. 3d, the RCS fluctuation of OAM waves with *l* = 1 is apparently different from that of the plane wave. At a specific angle when the RCS of the plane wave is at its peak, the RCS of the OAM wave with *l* = 1 is approximately at its valley. In the experiment, the RCS of an OAM wave with *l* = 2 is also slightly different from that of the plane wave.

The above experimental results show that the phase gradient of an OAM wave leads to different RCS values in a specific incident angle. This fact is expected to contribute to RCS diversity among different OAM waves, i.e., RCS diversity gain can be obtained by combining the backscatter waves of the different OAM waves.

### Experiment 3: Evaluation of the RCS diversity with back scattered waves

**Figure 4:**
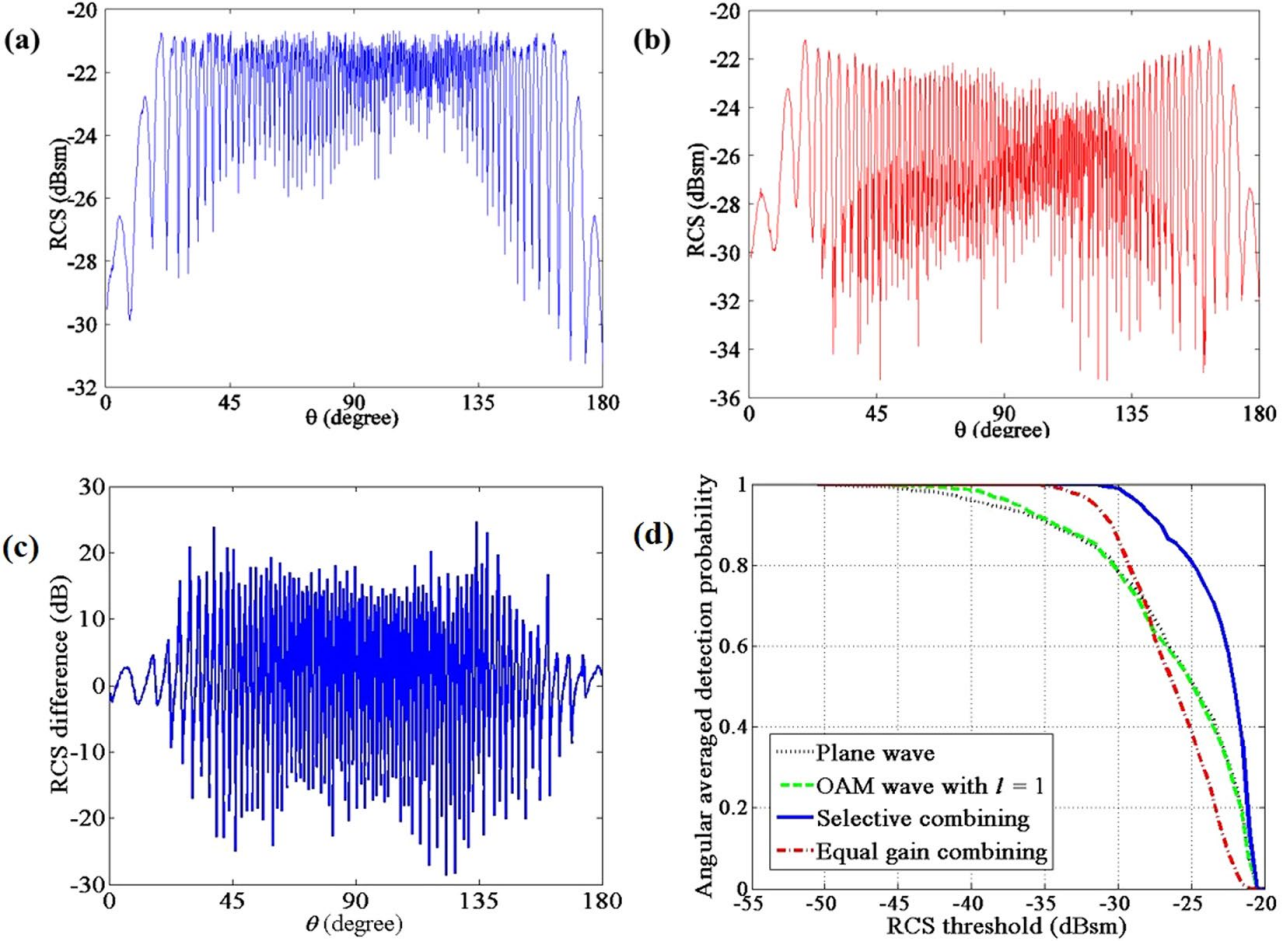
RCS diversity with combining schemes. (**a**) RCS diversity with equal combining. (**b**) RCS diversity with selective combining. (**c**) RCS difference of OAM with *l* = 1 to the plane wave. (**d**) Angular averaged detection probability versus RCS threshold according to the receiver sensitivity.

From the above experiments, the phase gradient produced by the OAM spiral spatial phase has a significant impact on the RCS of the complicated target. Compared with the plane wave, the difference is outstanding (see Figure S2 in the Supplementary Information). In this experiment, two combining methods are proposed to evaluate the RCS diversity, i.e., equal gain combining and selective combining. Figure 4 displays the results of the two combining schemes. As the OAM wave with *l* = 2 only has a slight RCS difference with the plane wave, only the plane wave and the OAM wave with *l* = 1 are taken into account in this experiment. Figure 4a shows the resultant RCS of equal gain combining, and Figure 4b shows that of the selective combining. In order to reveal the difference between RCS of OAM wave and plane wave in the same incident angle, Figure 4c illustrates the RCS difference of the OAM wave with *l* = 1 to the plane wave. The RCS difference fluctuates between −30 dB to 30 dB. Moreover, Figure 4d illustrates the angular averaged detection probability defined by the probability above the RCS threshold within 360°, which shows the averaged performance from all the incident angles. The plane wave, the OAM wave with *l* = 1 and the two combining schemes are presented. From the figure, both combining schemes improve the performance of weak RCS (RCS <−30 dBsm) in this experiment. As the selective combining scheme always selects the larger RCS from the plane wave and OAM wave, it has the best performance, i.e., more than 15 dB gain obtained compared with the plane wave. As shown in Fig. 4c, the averaged RCS difference within 180° almost approaches zero dB. Based on this fact, considering the symmetry of RCS within 360°, it is easy to understand that the statistical value such as the angular averaged detection probability of the OAM wave with the mode number *l* = 1 is similar to that of the plane wave, as indicated in Fig. 4d. However, after utilizing combining methods, especially selective combining, the angular averaged detection probability can be improved significantly.

### Experiment 4: RCS diversity of bi-static scattered waves

**Figure 5:**
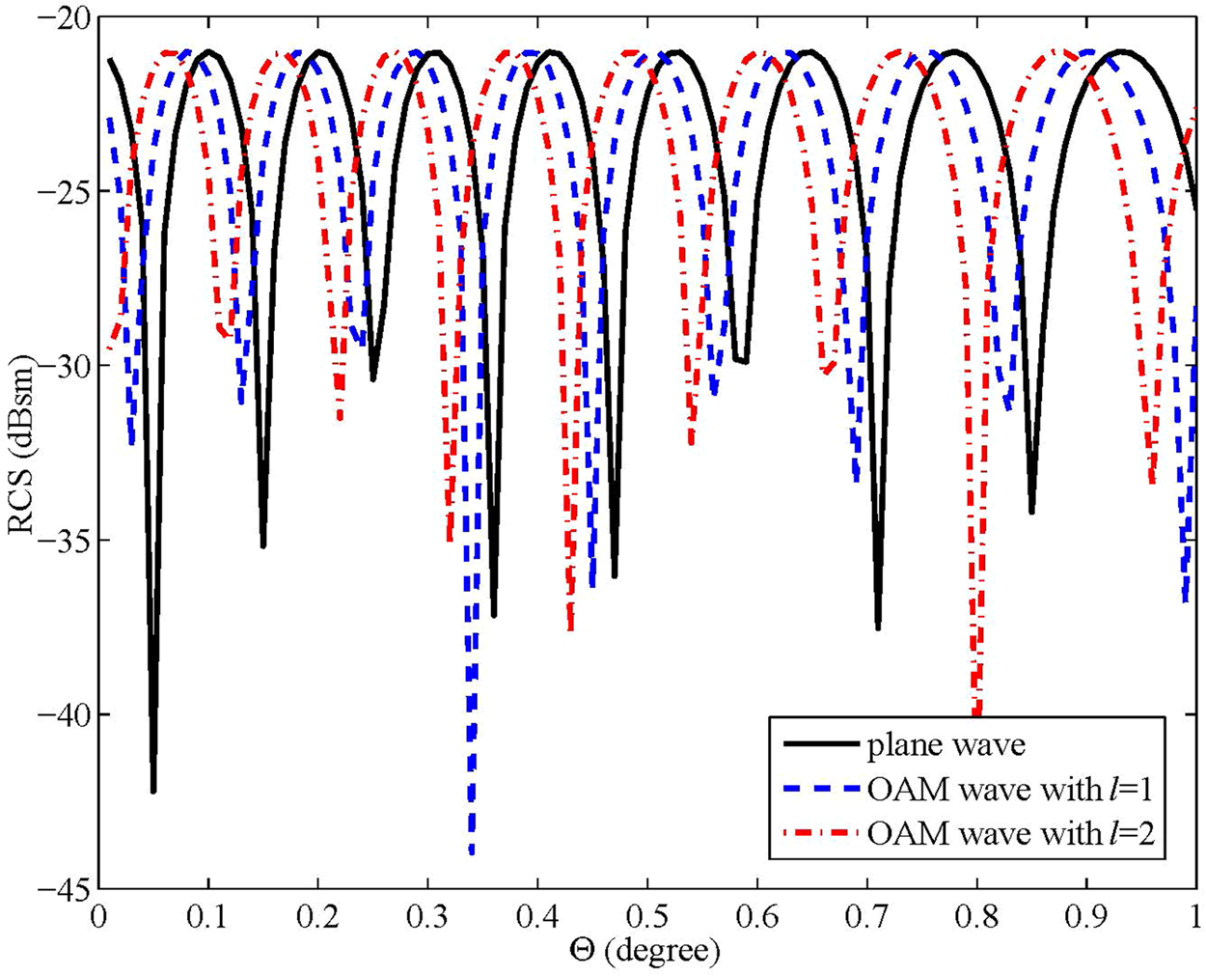
RCS of the bi-static scattered waves. The transmitting power is 10 dBm, the radius of the ball is 2.5 cm, the distance between balls is 12 cm, the distance between the antenna and target is 3 m, the wave frequency is 35 GHz, the height of the target is 90 cm, and the height of the antennas is 82 cm.

This experiment verifies that the RCS diversity not only occurs in monostatic backscattered waves but also in the bi-static scattered waves. Further simulation is processed in this experiment to show that the RCS diversity still holds for bi-static scatted waves. The simulation scenario and the major parameters are the same as the above experiments; the only difference is that the receiving antenna rotates around the target along a circle with radius *R* and the target does not rotate. The result is shown in Fig. 5, where Θ corresponds to the angle rotated between the transmitter and the receiver. Because the RCS fluctuates too frequently when the receiver rotates (see Figure S3a to S3c in the Supplementary Information), only a region with 1° is illustrated as an example to show the RCS difference. The peaks of the RCS values of different waves locate in different positions. Therefore, for bi-static scattered waves, OAM waves with different modes can also contribute to RCS diversity (see Figure S3d in the Supplementary Information).

## Discussion

In our experiments, in order to simplify the experiment, a simple target is composed of a single metal ball and the complicated target is composed of two metal balls. Other kinds of scatters can be adopted in this experiment, such as metal plates, corner reflectors, or even a scaled real target model. The antenna used to generate the OAM wave is a horn antenna with a SPP. However, other types of antennas, such as phased array, can also be used to generate OAM waves.

Only OAM waves with mode *l* = 1 and *l* = 2 are employed because the experimental distance is small, i.e., *d* = 3 m. The phase gradient can be easily detected. For a long distance, the OAM waves with a small mode number cannot provide a significant phase gradient. In this case, the OAM waves with large mode numbers can be employed. The simulation illustrated in Figure S4 in the Supplementary Information confirms this assertion. The positive and negative signs of mode numbers (i.e., topological charges) of OAM waves also have different spatial phase patterns with handedness. OAM waves with odd and even modes vary in RCS from different incident angles. This fact also holds for the OAM waves with different signs of the mode number. Due to the RCS difference, the selective combining can provide outstanding RCS performance. (For more explanation, refer to the simulation results revealed by Figures S5, S6 and S7 in the Supplementary Information).

The RCS diversity proven in our experiment implies that the OAM wave has the capability of anti-stealth applied in monostatic radar as well as bi-static radar. For example, some cognitive scheme can be designed for radar to identify the OAM wave with larger RCS. Selective combining can bring outstanding RCS improvement and increase the detection probability of the stealth target illuminated by the common plane wave.

## Method

In this experiment, OAM waves with different modes are obtained by SPP. The SPPs are made of polyethylene. To guarantee that the SPPs are able to generate the correct OAM modes, the SPPs are first measured in the planar near-field anechoic chamber. The rotary table has an angular resolution of 0.1°; the mechanical error of the experiment is at the order of 1 mm. All the measurements are based on an Agilent PNX Series Network Analyser 5244A.

## Electronic supplementary material

Supplementary Information
