# Supplementary material for: RCS Diversity of Electromagnetic Wave Carrying Orbital Angular Momentum
Source: Sci Rep. 2017 Nov 13;7:15412. doi: 10.1038/s41598-017-15250-7 (PMC5684226; doi:10.1038/s41598-017-15250-7)
Supplement: Supplementary file 1 — Supplementary Information [file 41598_2017_15250_MOESM1_ESM.pdf]

Supplementary Information

RCS Diversity of Electromagnetic Wave Carrying Orbital Angular Momentum

Chao ZHANG*, Dong CHEN, and Xuefeng JIANG

Labs of Avionics, School of Aerospace Engineering,
Tsinghua University, Beijing, 100084, P. R. China
*To whom correspondence should be addressed; E-mail: zhangchao@tsinghua.edu.cn

Comparison of experiments and simulations

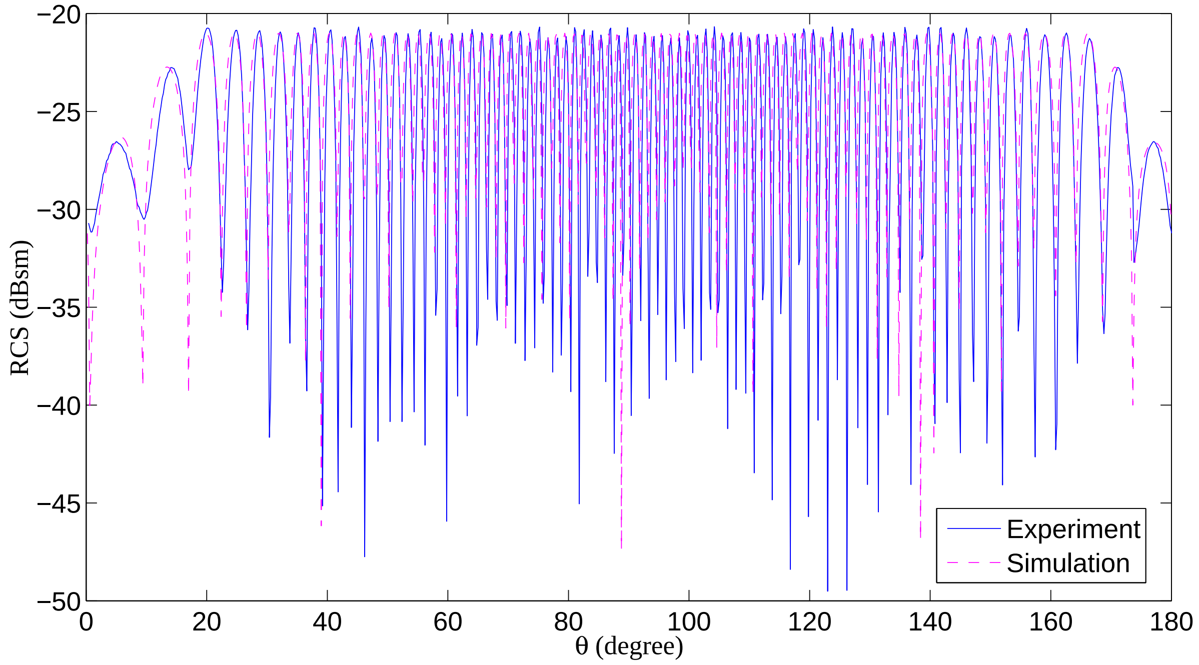

(a)

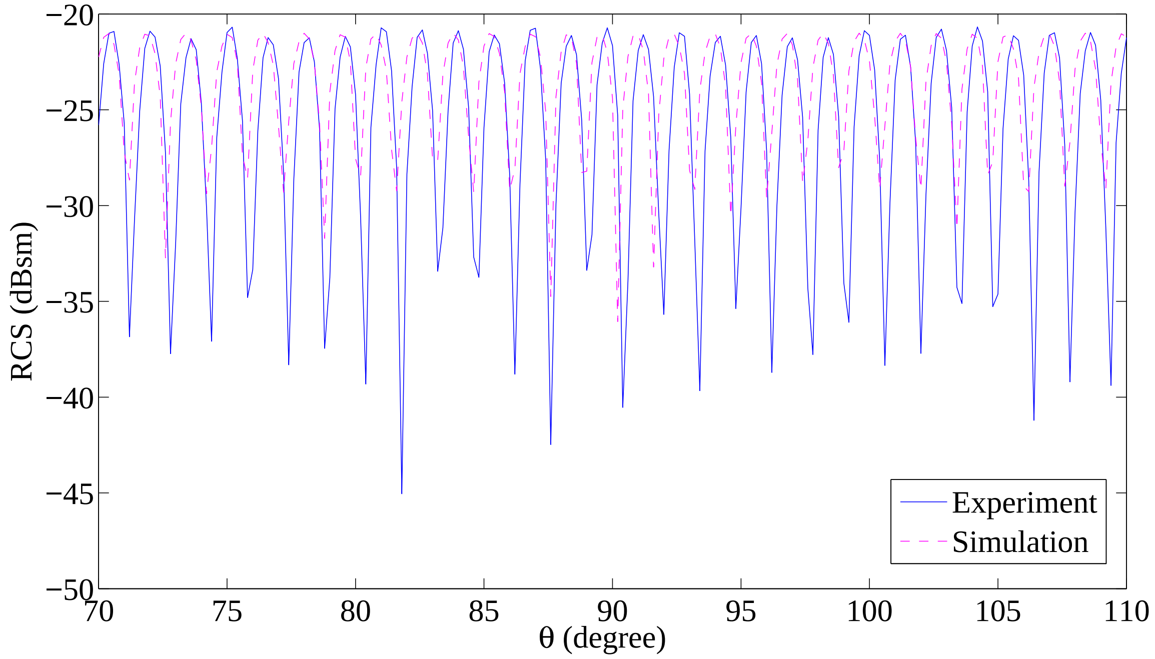
−20
−25
−30
−35
−40
−45
−50
RCS (dBsm)
70
75
80
85
90
95
100
105
110
θ (degree)
Experiment
Simulation

(b)

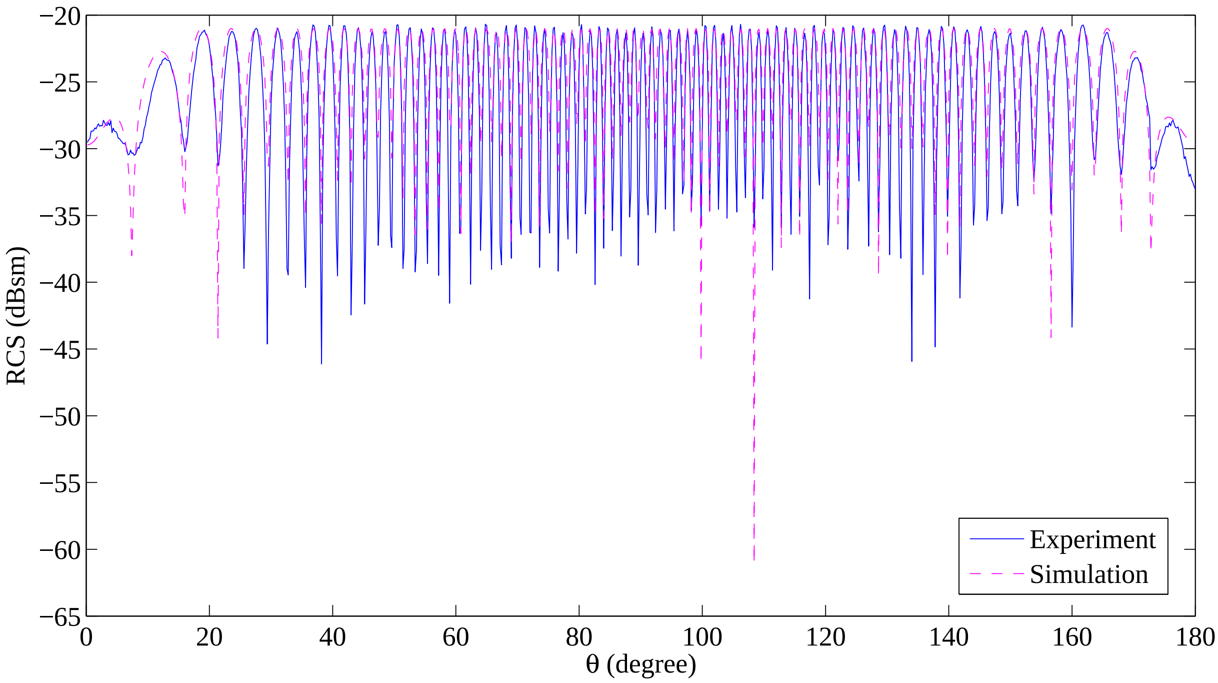
−20
−25
−30
−35
−40
−45
−50
−55
−60
−65
RCS (dBsm)
0
20
40
60
80
100
120
140
160
180
θ (degree)
Experiment
Simulation

(c)

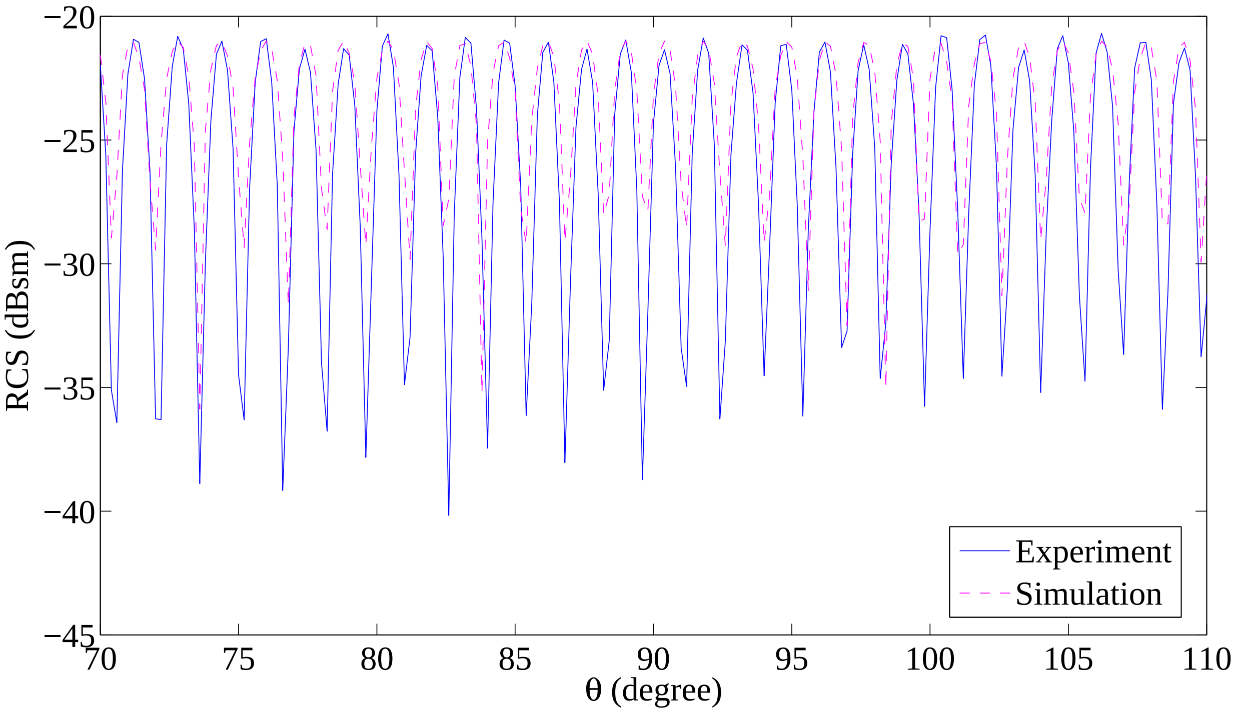
−20
−25
−30
−35
−40
−45
70
75
80
85
90
95
100
105
110
RCS (dBsm)
θ (degree)
Experiment
Simulation

(d)

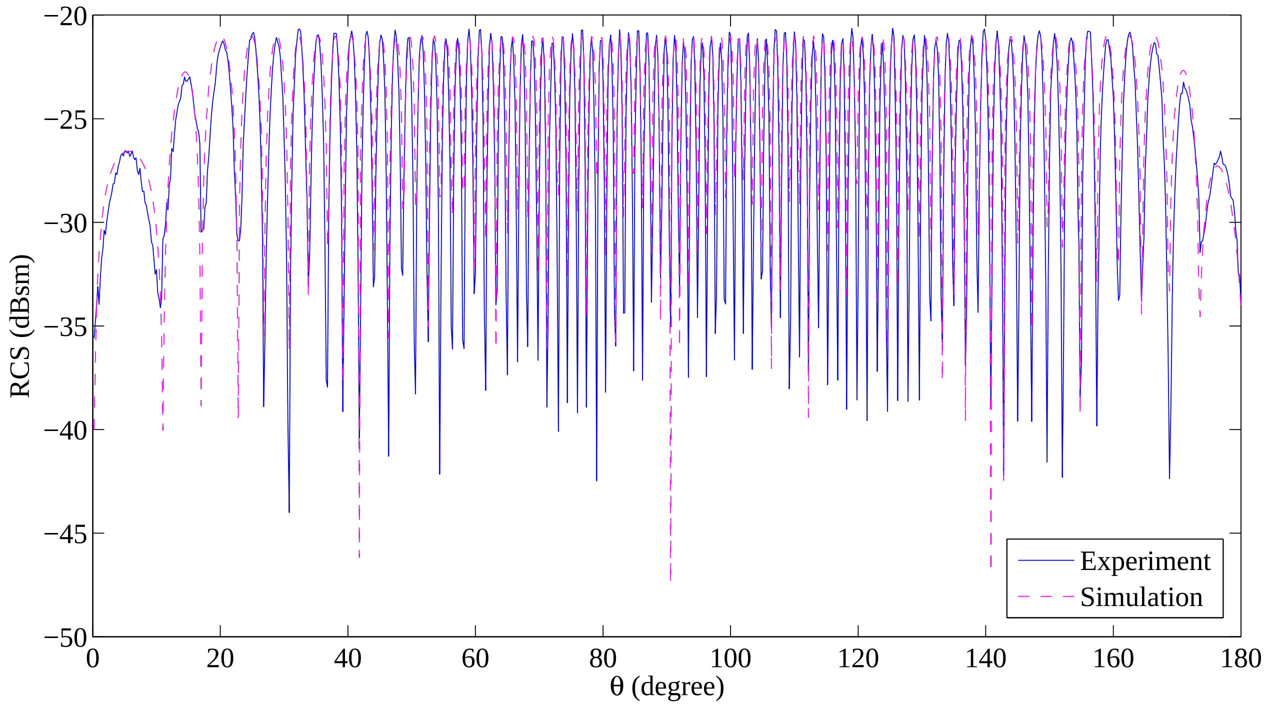
−20
−25
−30
−35
−40
−45
−50
0
20
40
60
80
100
120
140
160
180
RCS (dBsm)
θ (degree)
Experiment
Simulation

(e)

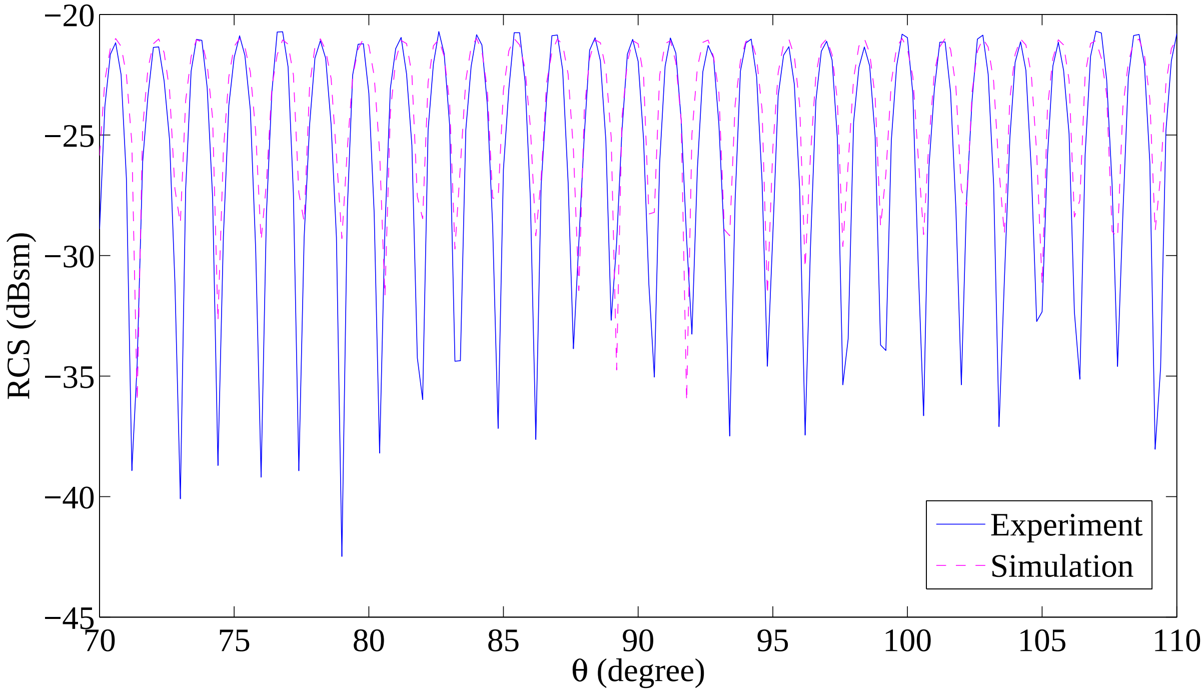

(f)

Figure |S1. Simulation results compared with experimental results. (a) Comparison of the RCS experiment and simulation by the plane wave. The θ varies from 0° to 180°. (b) To see the details, Figure S1a is zoomed, and the region of θ from 70° to 110° is highlighted. (c) Comparison of the RCS experiment and simulation by the OAM wave with l = 1. The θ varies from 0° to 180°. (d) To see the details, Figure S1c is zoomed, and the region of θ from 70° to 110° is highlighted. (e) Comparison of the RCS experiment and simulation by the OAM wave with l = 2. The θ varies from 0° to 180°. (f) To see the details, Figure S1e is zoomed and the region of θ from 70° to 110° is highlighted.

RCS difference evaluation

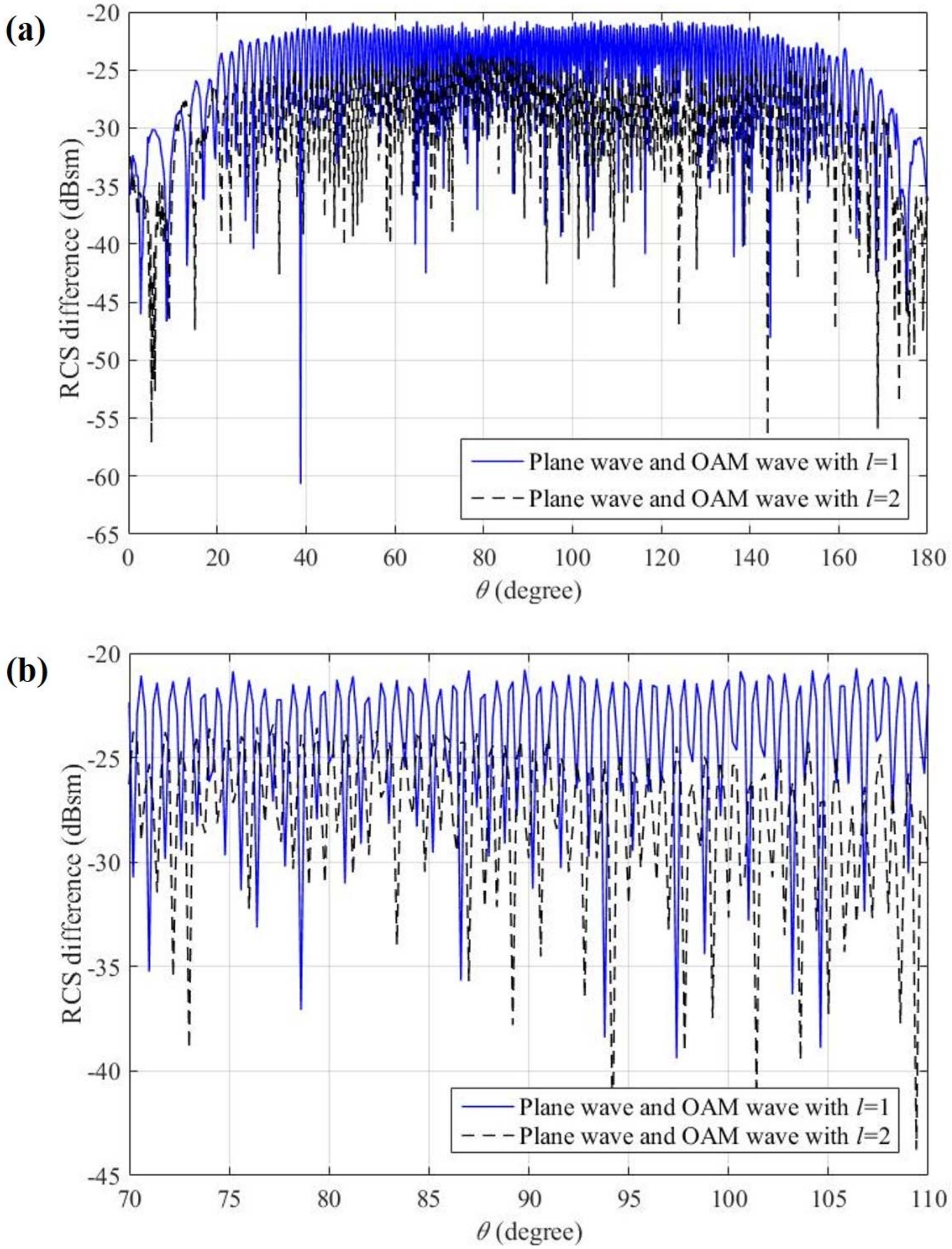

Figure |S2. RCS difference of plane wave and OAM waves. (a) RCS difference with θ varies from 0° to 180°, which is calculated from the data of Figure 3. (b) To see the details, Figure S2a is zoomed and the RCS difference with θ from 70° to 110° is highlighted.

RCS with bi-static scattered waves

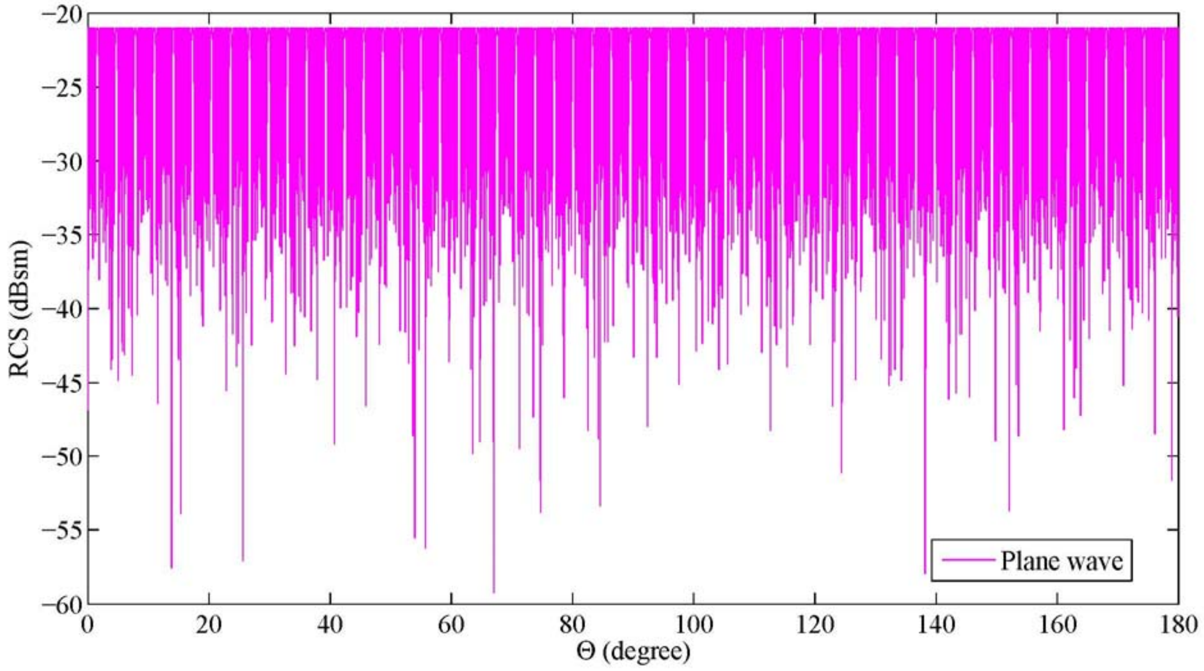

(a)

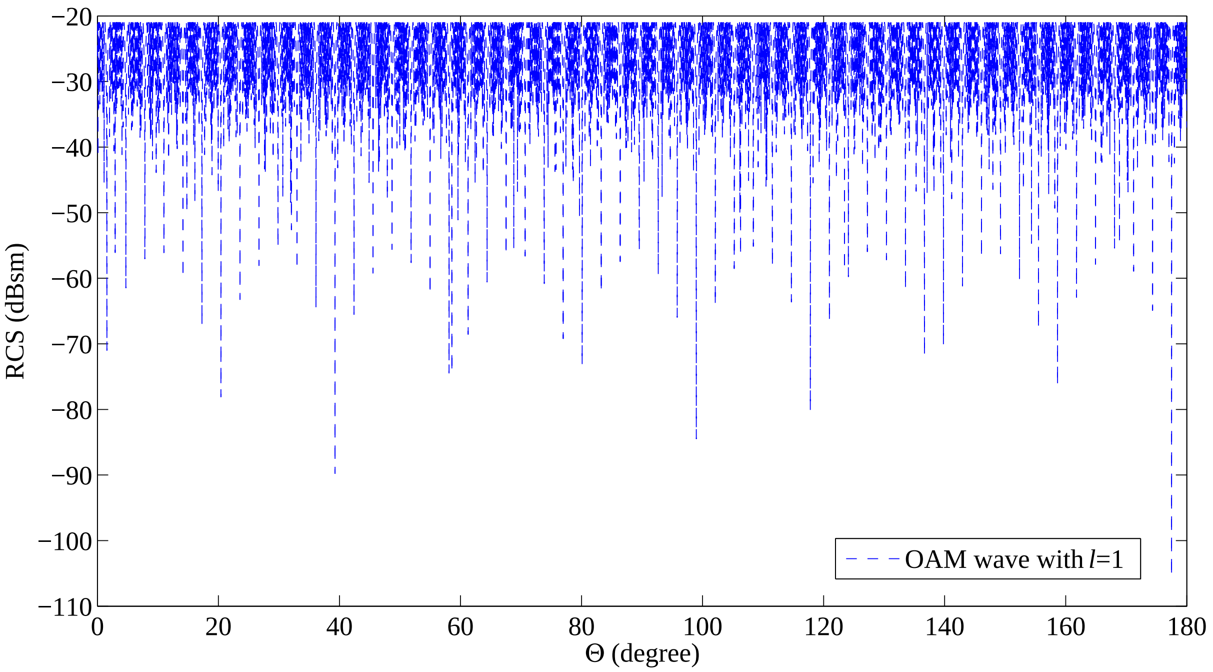

(b)

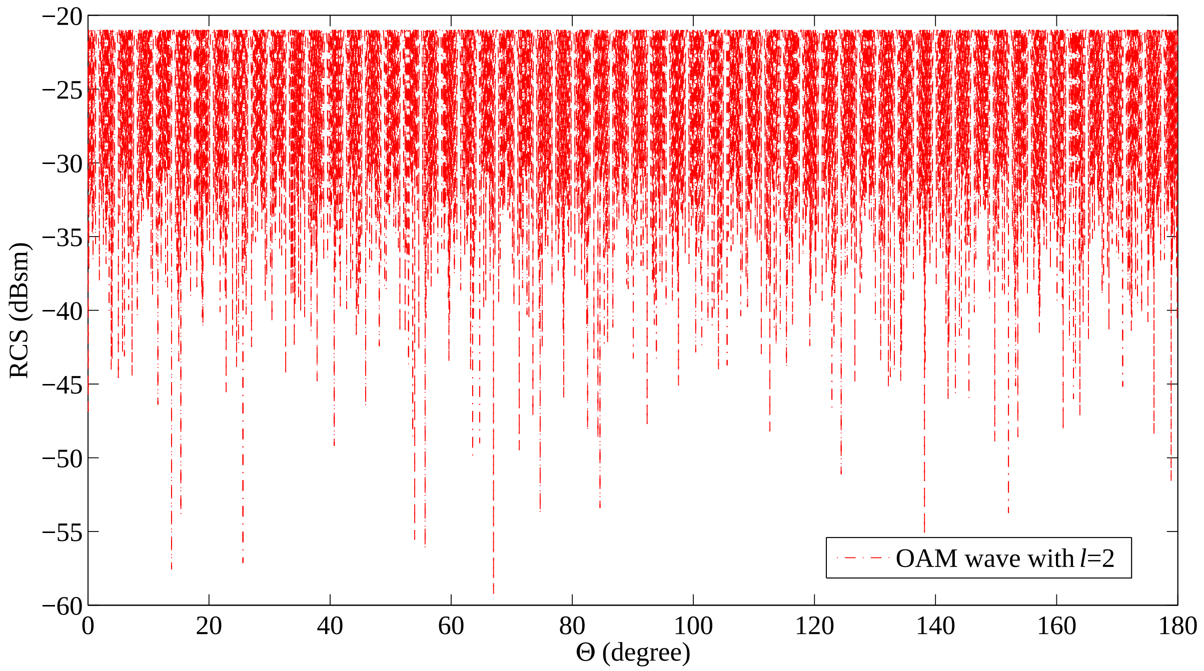
−20
−25
−30
−35
−40
−45
−50
−55
−60
RCS (dBsm)
0
20
40
60
80
100
120
140
160
180
Θ (degree)
OAM wave with l=2

(c)

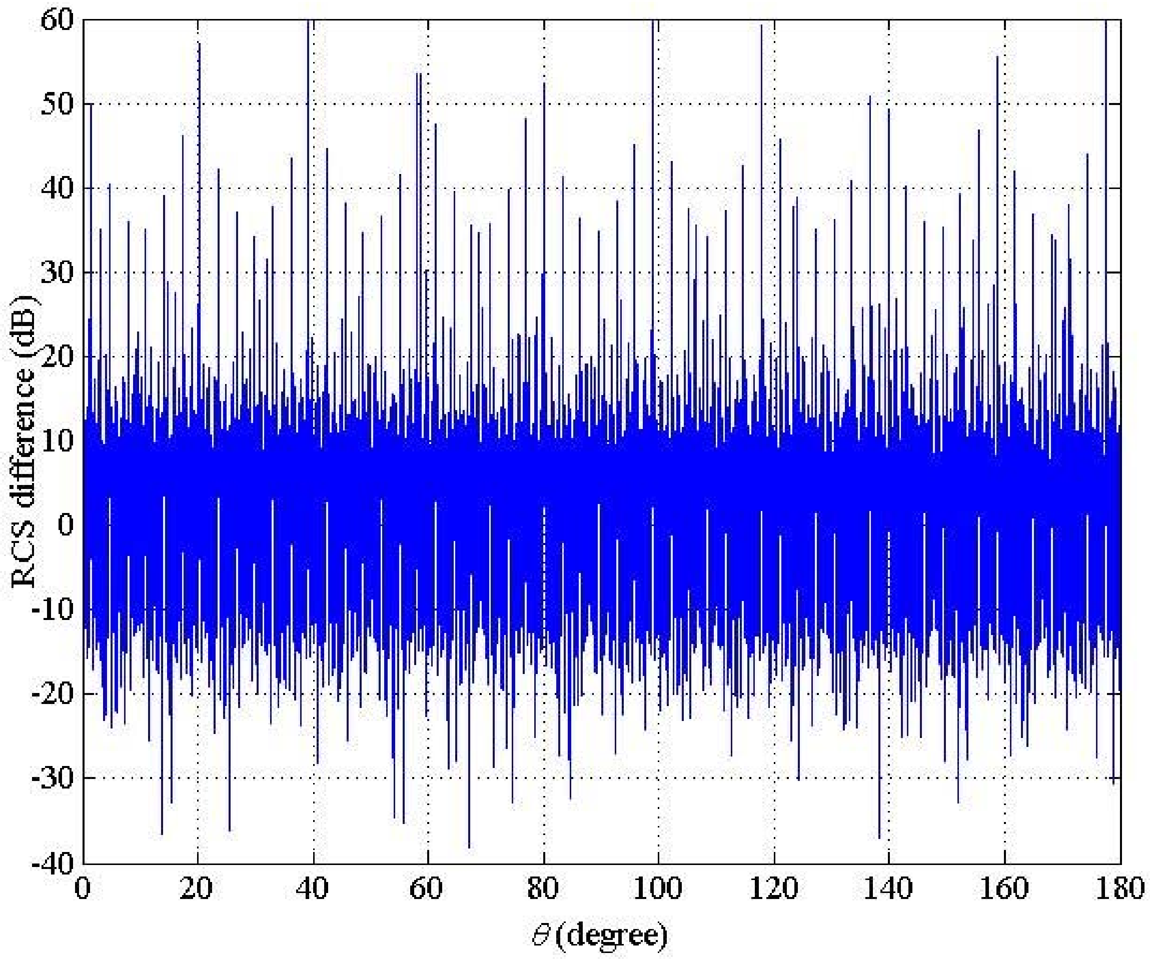
60
50
40
30
20
10
0
-10
-20
-30
-40
RCS difference (dB)
0
20
40
60
80
100
120
140
160
180
θ (degree)

(d)

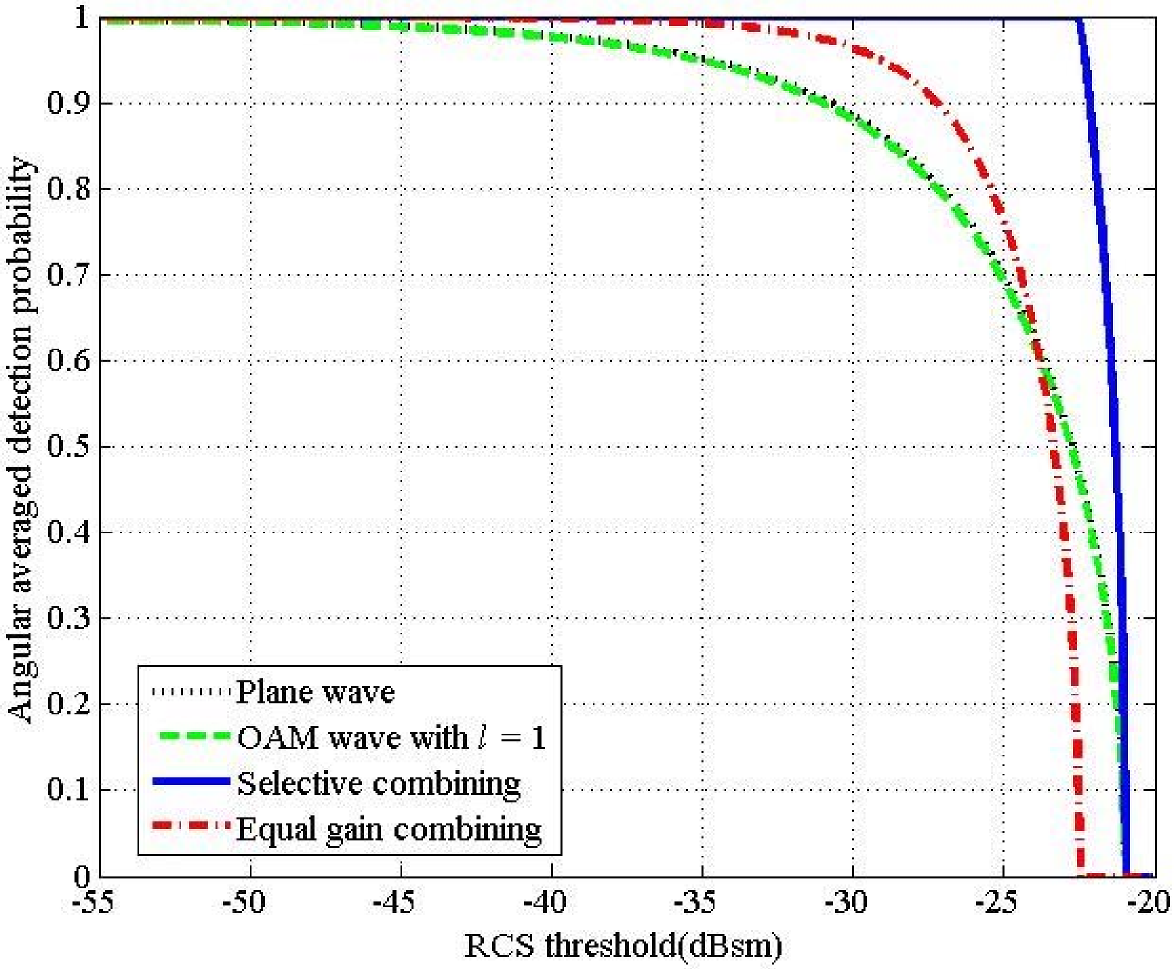

(e)

Figure |S3. RCS and detection probability simulation of OAM waves for bi-static scattered waves. (a) Plane wave. (b) OAM wave with l = 1. (c) OAM wave with l = 2. (d) RCS difference of OAM wave with l=1 to the plane wave. (e) Angular averaged detection probability versus the RCS threshold according to the receiver sensitivity.

Simulation with large OAM modes

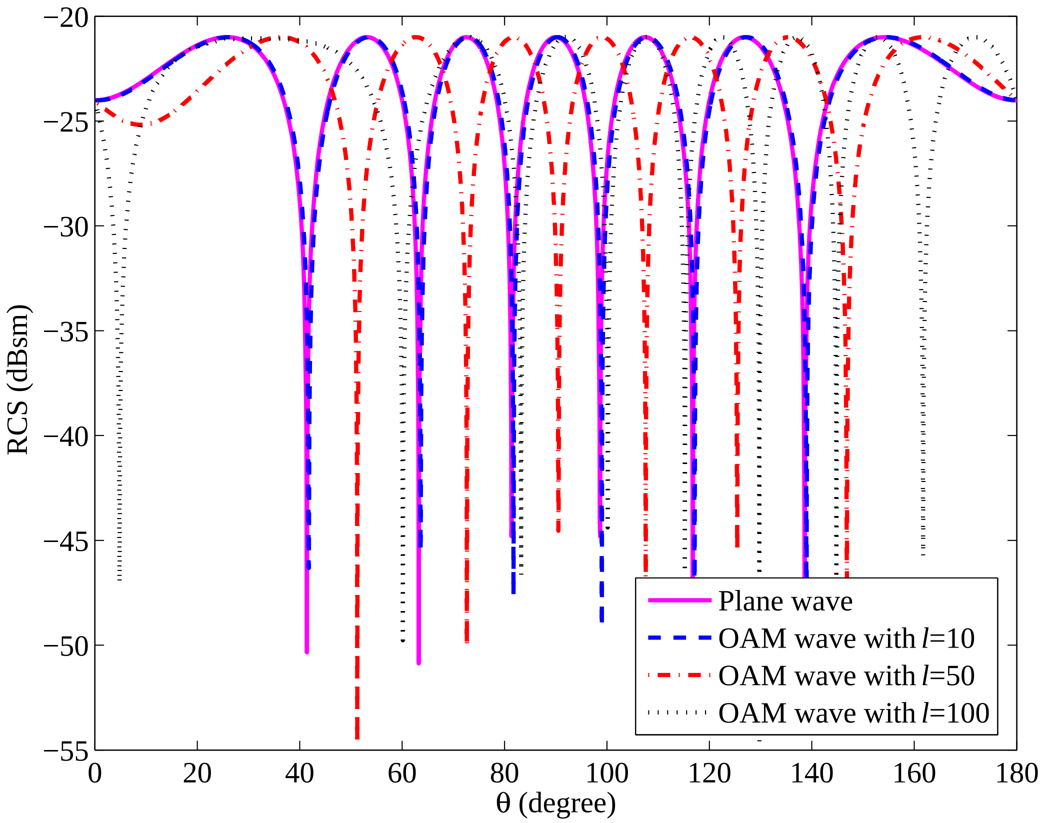

Figure |S4. Simulation of OAM wave with a large mode number. This simulation scenario with d = 1500 m, the interval of the two scatter points is 0.1 m, the two scatter balls are deployed in the transverse section of the ring beam of an OAM wave. The beam angle is assumed to be 5°. As indicated in the figure, in long-distance detection, a larger phase gradient is required. Therefore, the OAM wave with a large mode number can be employed to produce significant RCS diversity.

Simulation with Odd and Even OAM modes

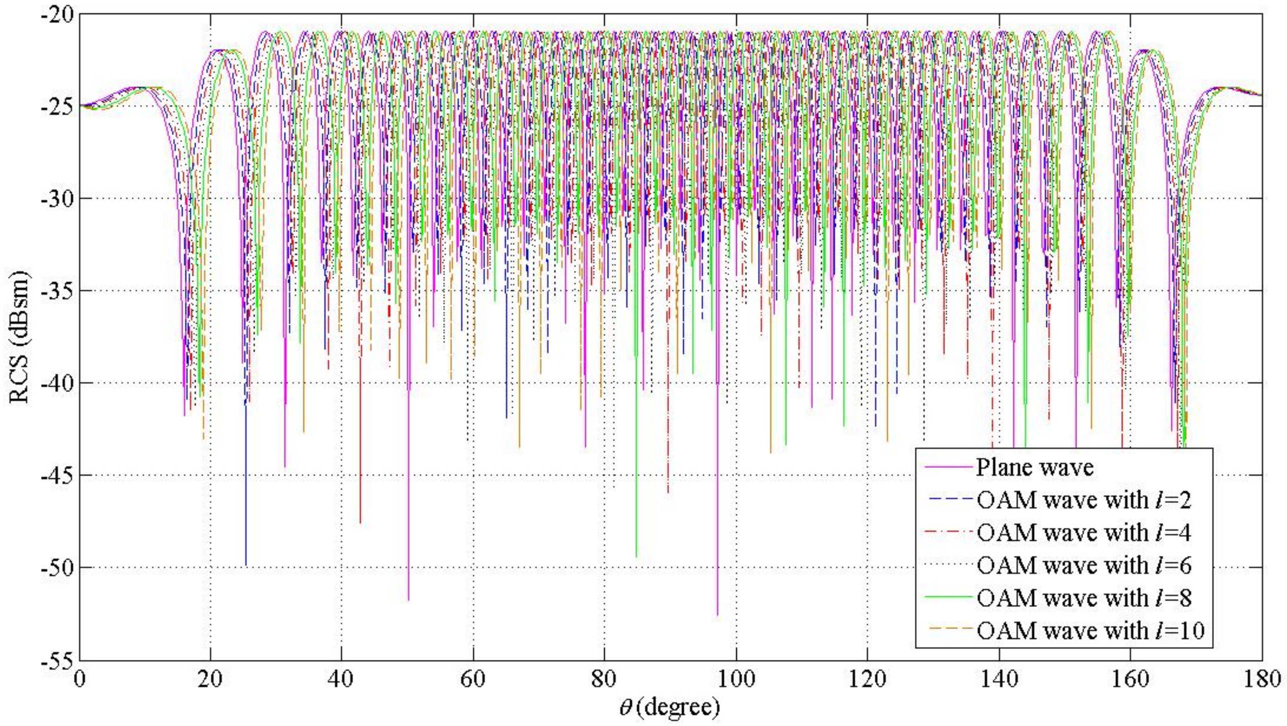

(a)

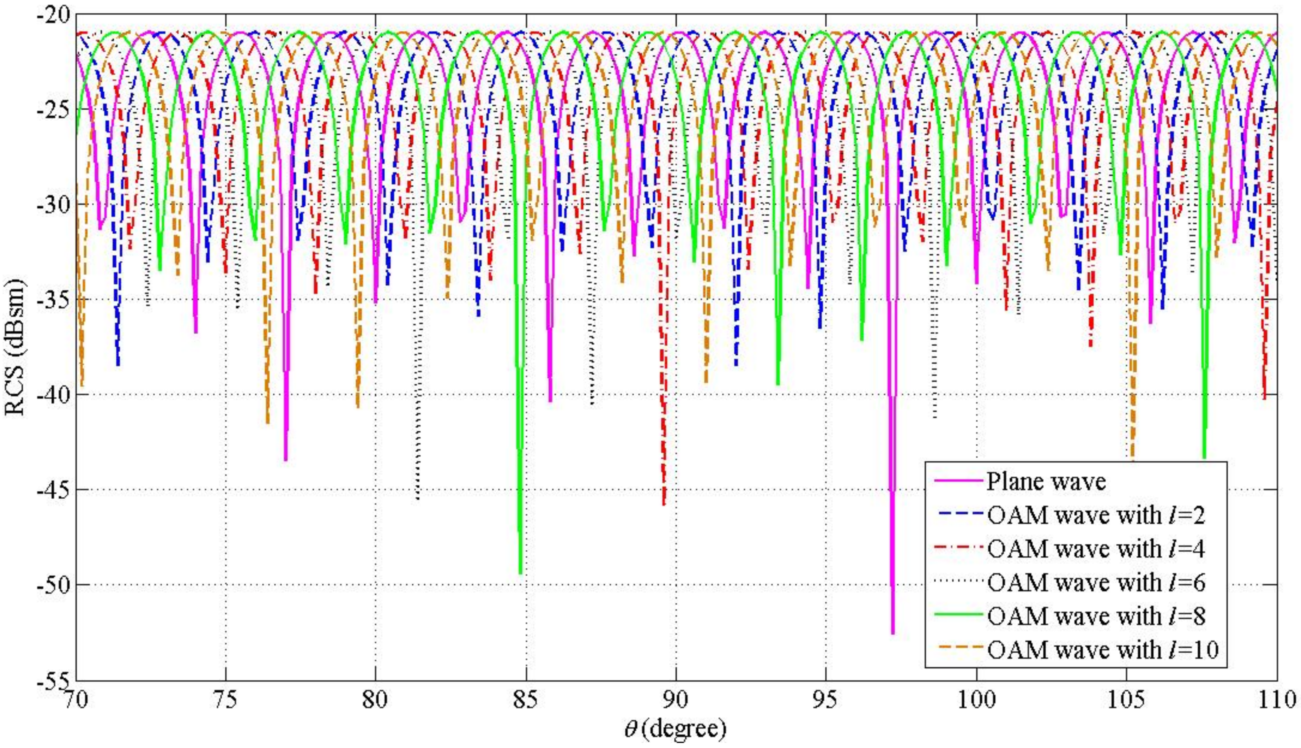

(b)

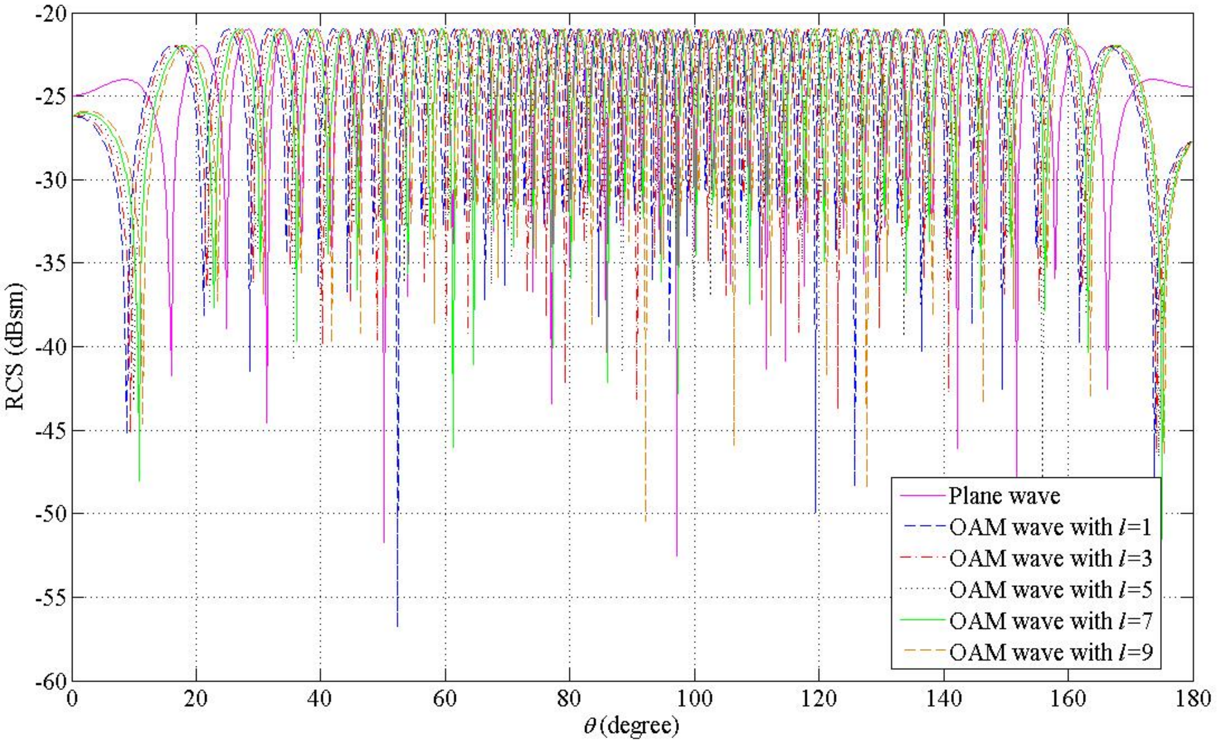

(c)

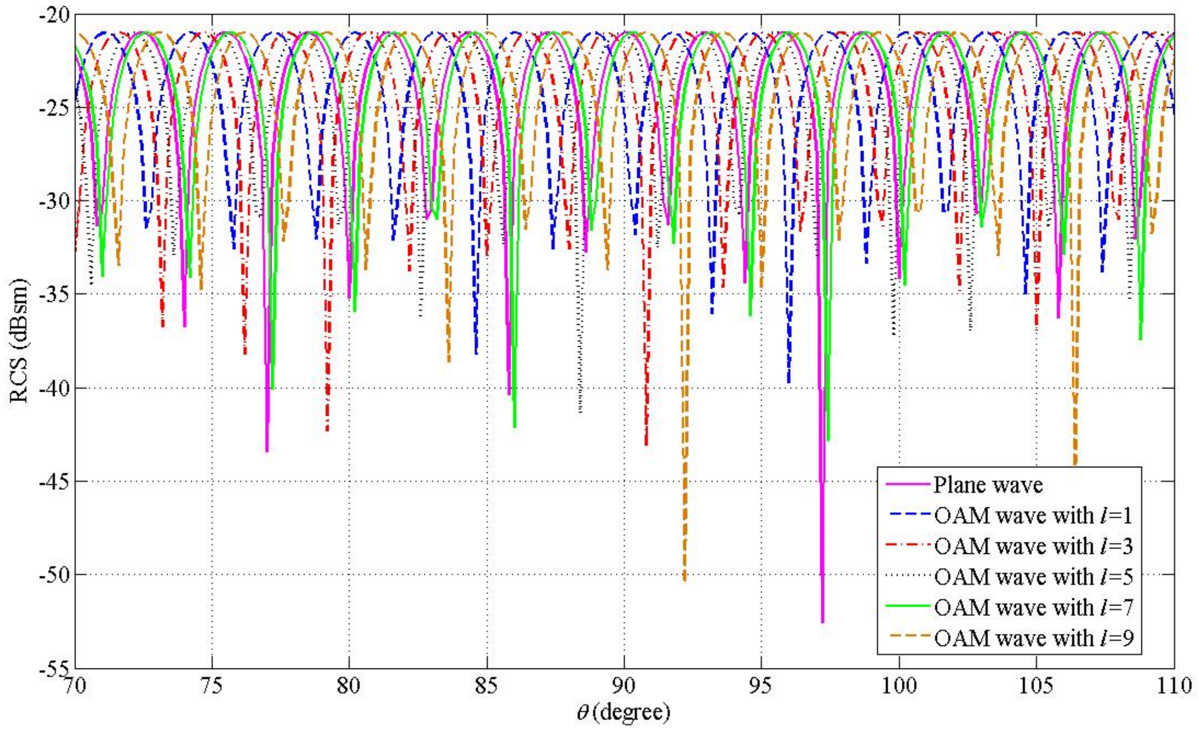

(d)

Figure |S5. Simulation with odd and even OAM modes. This simulation scenario with d = 3 m, one scatter locates at the centre, and the other scatter rotates around the former with radius r = 6 cm. The scatters are 0.2 m higher than the antennas. Other parameters are the same as Figure S1. OAM waves with positive and negative mode numbers (i.e., topological charges) are compared. (a) Comparison of RCS with plane wave, OAM wave with even numbers. (b) To see the details, Figure S5a is zoomed and the RCS difference with θ from 70° to 110° is highlighted. (c) Comparison of RCS with plane wave, OAM wave with odd numbers. (d) To see the details, Figure S5c is zoomed, and the RCS difference with θ from 70° to 110° is highlighted.

Simulation with Positive and Negative OAM modes

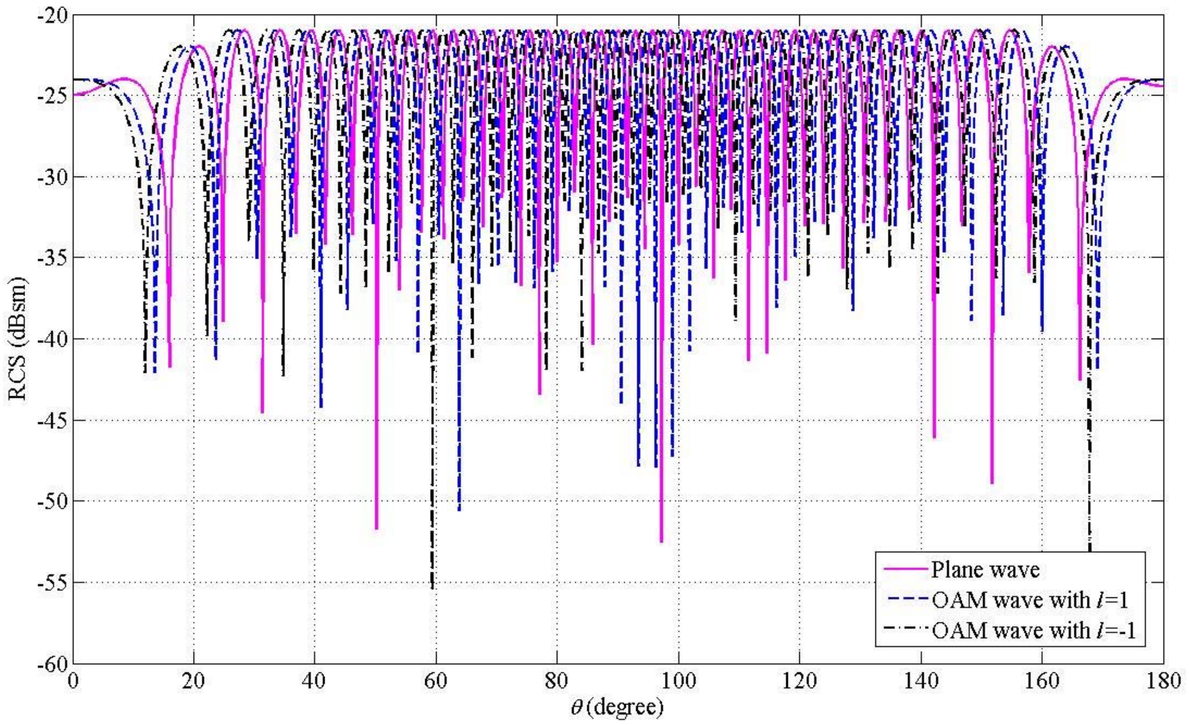

(a)

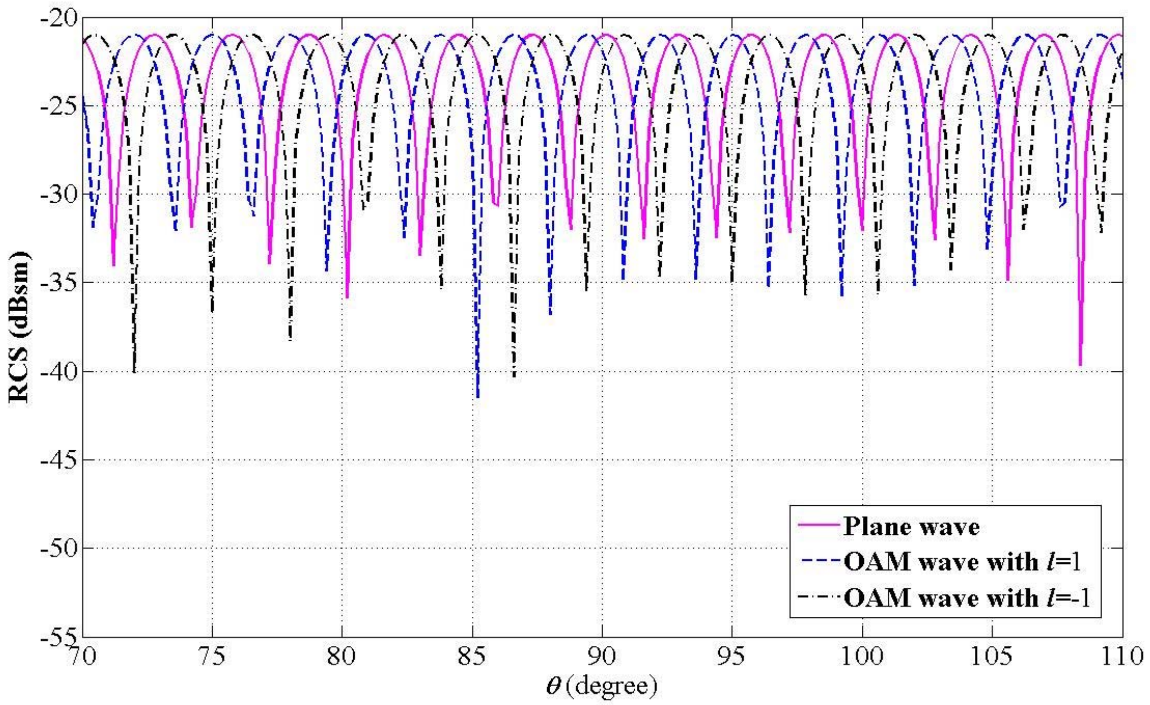

(b)

Figure |S6. Simulation with positive and negative OAM modes. The simulation parameters are the same as the simulation in Figure S5. OAM waves with positive and negative mode numbers (i.e., topological charges) are compared. (a) Comparison of RCS with plane wave, OAM wave with *l* = 1 and OAM wave with *l* = -1. (b) To see the details, Figure S6a is zoomed, and the RCS difference with θ from 70° to 110° is highlighted.

Angular Averaged Detection Probability with More OAM modes

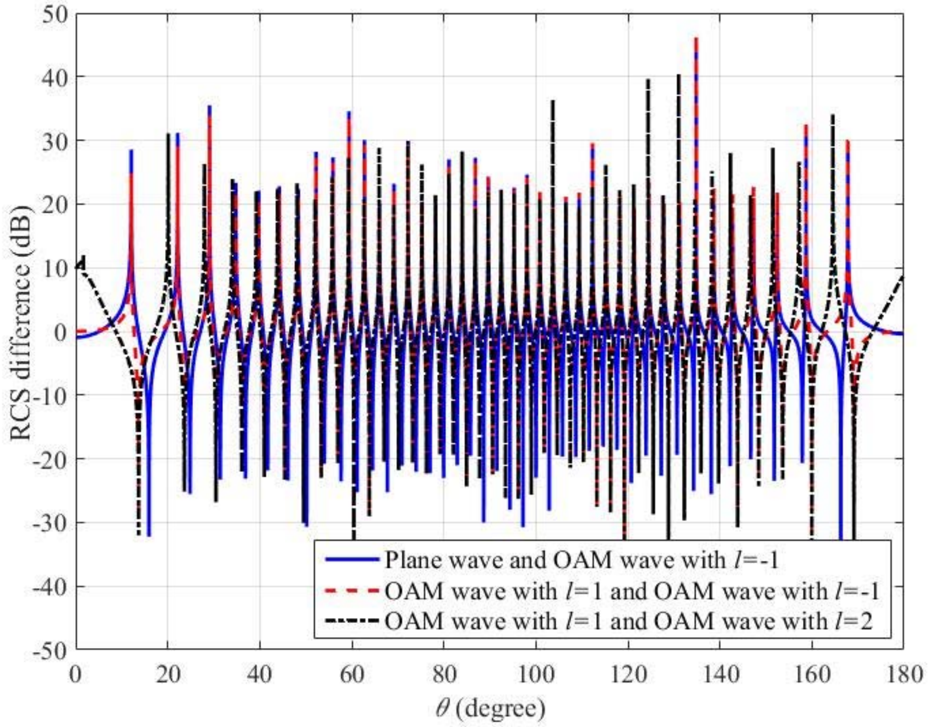

(a)

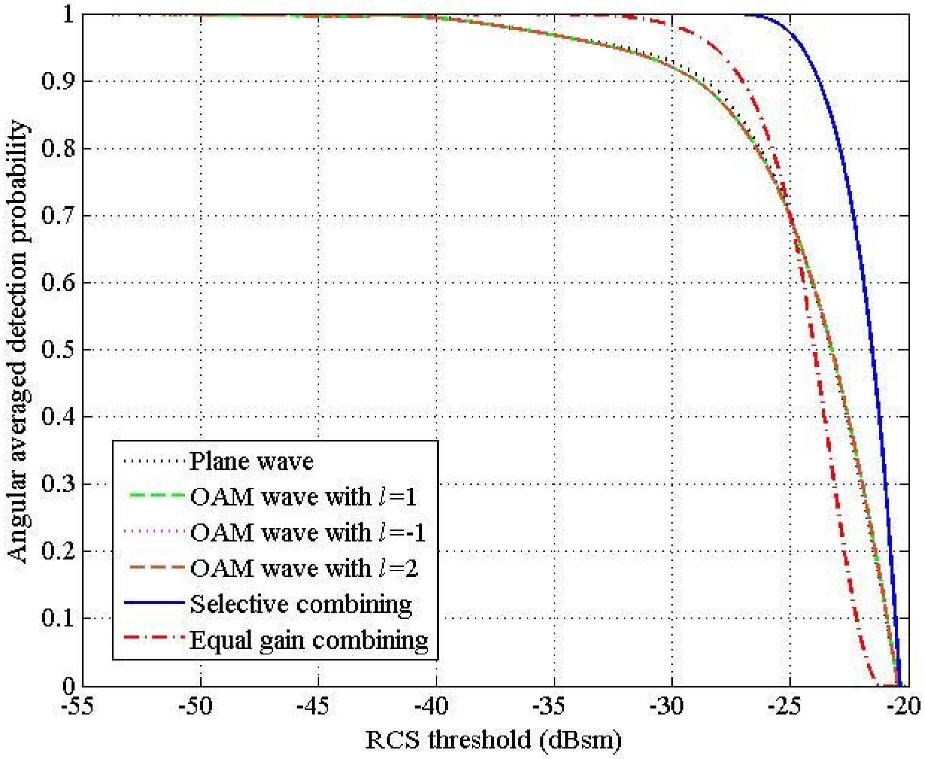

(b)

Figure |S7. Angular averaged detection probability versus RCS threshold according to the receiver sensitivity. More cases listed in Fig. S5 and Fig. S6 are included for comparison. In order to investigate the performance of OAM wave with odd and even OAM mode, as well as the positive and negative modes, the angular averaged detection probability of OAM waves with l=-1, 1, 2 are compared. (a) The RCS difference of the OAM waves and plane wave is notable. (d) The selective combining performs outstandingly due to the capability of RCS diversity.
